# A Comprehensive Evaluation of Iris Segmentation on Benchmarking Datasets

**DOI:** 10.3390/s24217079

**Published:** 2024-11-03

**Authors:** Mst Rumana Sumi, Priyanka Das, Afzal Hossain, Soumyabrata Dey, Stephanie Schuckers

**Affiliations:** 1 Department of ECE, Clarkson University, Potsdam, NY 13699, USAafhossa@clarkson.edu (A.H.); sschucke@clarkson.edu (S.S.); 2 Department of Computer Science, Clarkson University, Potsdam, NY 13699, USA

**Keywords:** biometric, iris, segmentation, deep learning

## Abstract

Iris is one of the most widely used biometric modalities because of its uniqueness, high matching performance, and inherently secure nature. Iris segmentation is an essential preliminary step for iris-based biometric authentication. The authentication accuracy is directly connected with the iris segmentation accuracy. In the last few years, deep-learning-based iris segmentation methodologies have increasingly been adopted because of their ability to handle challenging segmentation tasks and their advantages over traditional segmentation techniques. However, the biggest challenge to the biometric community is the scarcity of open-source resources for adoption for application and reproducibility. This review provides a comprehensive examination of available open-source iris segmentation resources, including datasets, algorithms, and tools. In the process, we designed three U-Net and U-Net++ architecture-influenced segmentation algorithms as standard benchmarks, trained them on a large composite dataset (>45K samples), and created 1K manually segmented ground truth masks. Overall, eleven state-of-the-art algorithms were benchmarked against five datasets encompassing multiple sensors, environmental conditions, demography, and illumination. This assessment highlights the strengths, limitations, and practical implications of each method and identifies gaps that future studies should address to improve segmentation accuracy and robustness. To foster future research, all resources developed during this work would be made publicly available.

## 1. Introduction

A traditional iris recognition system includes four basic steps: [1]—(1) iris image acquisition, (2) segmentation, (3) normalization, and (4) feature extraction and matching. Iris segmentation involves locating and isolating the iris from other regions of the eye, including noise detection like occlusion from eyelashes, eyelids, or hair. Thus, it plays an important role in the recognition system. When the iris segmentation fails, even the best feature extraction method will be incapable of generating an iris code that corresponds to the actual iris texture, increasing the chances of false rejection [2]. Iris image quality and acquisition conditions generally affect the accuracy of this segmentation task. An iris image captured in less-constrained environments may include noises, such as motion blur, eyelids or eyelash occlusion, eyeglass occlusions, off-angle iris, specular reflections, etc., that make iris segmentation more challenging.

Conventionally, iris images are captured under NIR illumination for biometric recognition. However, there is an interest in the community to develop iris recognition technologies under visible illumination (VIS) [3,4]. Traditional NIR-based iris segmentation methods are mostly based on Daugman’s integro-differential operator [5], Wildes’ canny operator, or Hough transform [6]. These methods are suitable for cooperative or highly controlled iris biometric setups such as close acquisition distance, stop-and-stare verification, image capture under near-infrared illumination, etc. Traditional methods are not suitable for noisy data and data captured under a non-cooperative environment or less controlled environments such as acquisition at a distance, on the move, and image capture under visible illumination [7].

With the rapid increase of biometric recognition applications, there is a demand to update iris recognition technology, making it more noise-tolerant and user-friendly. Recently, deep learning (DL)-based iris segmentation methods have been introduced in research studies to overcome the shortcomings of traditional approaches [1,2,7,8]. However, open-source DL-based iris segmentation models are sparse and have limited reproducibility, limiting usage by the community and continuity for further improvement. Furthermore, one crucial requirement for developing efficient DL models is the availability of accurate ground truth iris masks in large volumes, and the unavailability of that is a significant limitation. Hofbauer et al. [9] made strides by publicly releasing two ground truth datasets, IRISSEG-CC [10] and IRISSEG-EP [11]. Nonetheless, a notable drawback of these datasets is their omission of eyelash annotations, which compromises the ability of models trained on them to detect eyelash occlusions, adversely affecting the performance of iris recognition systems.

In the literature, several iris segmentation techniques have been proposed, with reported success and limitations. However, some of the challenges, which are yet to be addressed, are listed below:

- Datasets used for the development of algorithms are often proprietary and lack crucial labels for features such as eyelashes, which are essential for accurate segmentation.
- The scarcity of open-source codes hinders the reproducibility of these methods.
- Algorithms are often evaluated on the same dataset they were trained on, limiting cross-dataset performance analysis, which is crucial for generalization and robustness in real-world applications.

This study attempts to identify the gaps in resources for iris segmentation and benchmark the open-source algorithms (both traditional and DL-based) against common datasets for direct comparison. In the process, the performance of the algorithms is evaluated based on several criteria:

- **Sensitivity:** The algorithms are assessed for their sensitivity toward different sensors, environments, and demographic variations. This involves a multi-dataset evaluation to understand how well the algorithms can adapt to various conditions.
- **Generalization capability:** The cross-dataset performance of the algorithms is examined to determine how well an algorithm can generalize its learning from one dataset to another, which may have different characteristics.
- **Practicability:** The practical aspects of the algorithms are also evaluated, including the inference time, the number of parameters, and the size of the model. These factors contribute to the feasibility of deploying the algorithm in real-world applications.
- **Noise detection capability:** The ability of the algorithms to detect and handle noise, such as eyelash detection, is assessed. This capability is important for improving the accuracy of the algorithms.
- **Illumination:** The performance of the algorithms under different lighting conditions, such as Near-Infrared (NIR) and Visible Spectrum (VIS), is evaluated. This helps in understanding the versatility and robustness of the algorithms in varying illumination scenarios.

In addition to the thorough comparative analysis of the open-source iris segmentation algorithms, we developed an end-to-end iris segmentation resource set with the motivation to append to the fragmented existing public resources. The resource set consists of the following: 1. A *composite iris dataset of 45k+ samples*, gleaning publicly available datasets along with corresponding iris masks generated using OSIRISv4.1 (Open Source Iris Recognition Software) [12]; 2. A GUI-based *iris segmentation toolkit* to generate iris masks (ground truth); 3. A set of *1K challenging ground truths*, including labels for eyelashes, hand-crafted using the GUI; and 4. *Three iris segmentation models* influenced by U-Net architecture trained on the composite dataset and fine-tuned on the handcrafted ground truth. The main contributions of our study are as follows:

- **Benchmarking of eleven iris segmentation methods** against **five datasets** of different compositions (illumination, sensor, noise level, demography), providing consistent and fair comparisons that offer valuable insights into real-world performance.
- **Development of a manual segmentation toolkit** and preparation of **1k manually segmented iris masks** of challenging samples such as iris images with eyelashes, eyeglasses, eyelid occlusion, and low usable iris area.
- **Development of three iris segmentation benchmarked models** influenced by state-of-art U-Net [13] and U-Net++ [14] architectures (average accuracy >90%), have generalized learning ability (cross-dataset accuracy >90%), are efficient in detecting eyelashes (average accuracy >90%), and perform equally well on NIR and VIS datasets.
- **Open-source** the resource set for the benefit of the research community, with the exception of the composite dataset for licensing restrictions. However, the composite dataset is reproducible—the datasets are publicly available, and OSIRIS is open-source. The resource set is available at https://github.com/RumanaSum/Iris-Segmentation (accessed on 24 February 2024).
- A comprehensive evaluation that highlights the strengths and limitations of the models, establishing a standard foundation for future research in iris segmentation and facilitating the development of highly accurate and robust benchmark models to serve as reliable references for further advancements in the field.

The rest of the paper is organized as follows: Section 2 introduces the related work on iris segmentation. Section 3 provides dataset preparations and implementation details of the developed models. Section 4 presents the results and analyzes the performance of the implemented methods. Section 5 discusses the limitations and road map for iris segmentation. The glossary section provides the glossary of terms used in this paper.

## 2. State of the Art

Multiple DL-based iris segmentation models have been proposed in the recent past. Following an assessment of the relevant work, the limitations in the scope of iris segmentation, such as availability of the models for reusability or reproducibility, availability of ground truth data for re-training, and eyelash detection capability, are summarized in Table 1.

Bezerra et al. [1] proposed GAN and a FCN-based iris segmentation method and evaluated them on NIR and VIS datasets. The source code or network weights of these models are not open-source. The authors publicly shared manually annotated 1K images from CASIA Thousand [25], 1K from CrEye-Iris [26], and 431 from MICHE-I [27] datasets. Lozej et al. [18] trained a U-Net [13]-based model on 200 manually labeled ground truths from the CASIA Interval dataset [25]. While the model weights are publicly available, the ground truth remains proprietary. Wang et al. [7] proposed a multi-tasking U-Net segmentation model that can generate iris masks, iris outer boundary, and pupil masks from the raw iris image. For training this model, the authors employed publicly available iris masks along with self-annotated pupil masks and iris outer boundary masks. The model is evaluated on CASIA Distance [25], UBIRIS.v2NICE.I [28], and MICHE-I [27] datasets. Their self-annotated ground truth masks and network weights are publicly available. Trokielewicz et al. [24] fine-tuned a SegNet architecture with 1300 self-annotated postmortem iris images obtained from the Warsaw-BioBase-Post-Mortem-Iris v1.0 [29] dataset. Their annotated ground truths and source code were also released for further experimentation. Chen et al. [22] independently trained a CNN-based model on the CASIA Interval [25] and IIT Delhi Iris [30] datasets with self-annotated and publicly available ground truths (obtained from the IRISSEG-EP dataset). The self-annotated ground truths and the models are not public. Hofbauer et al. [9] publicly shared two ground truth datasets—IRISSEG-CC [10] and IRISSEG-EP [11]. One limitation of these datasets is that the eyelashes are not marked. Thus, the models trained on these datasets would lack the capability to detect eyelash occlusions, resulting in a negative impact on the performance of the subsequent iris recognition system. The authors [22] cautioned of misleading higher segmentation accuracy using the IRISSEG-EP [9] dataset as it excludes eyelash labels. A few other proposed iris segmentation studies provide no information regarding the availability of self-annotated ground truths, models, or network weight [8,15,16,17,19,20]. Huo et al. [8] proposed an Attention Mechanism U-Net++, employing a pruning scheme to obtain four iris segmentation networks. Wang et al. [16] developed a lightweight, FCN-based network. They adopted multi-level feature-dense fusion modules, multi-supervised training of multiscale images, and generative adversarial networks to improve the segmentation performance. Meng et al. [17] combined Swin-T with CNNs and introduced a bilateral segmentation backbone network. Zhang et al. [19] combined the dilated convolution with the U-Net to extract more global features. Jalilian et al. [20] proposed three types of FCN-based networks. Miron et al. [15] proposed a modified lightweight U-Net-based architecture. Lian et al. [31] added an attention module to the U-net to increase the weight of the iris. Despite the importance of eyelash labels on the training mask, only a few models in the literature [1,7,18,23] were trained with eyelash labels. The models proposed in [2,19,20] were trained on a public ground truth dataset [9], which does not have annotated eyelashes. The source of ground truth for training other models [8,15,16,17] was not mentioned; eyelash annotations were also not found by our visual inspection. The availability of more annotated iris masks with eyelash labels will strengthen the iris segmentation research. The review of related works also indicates that a thorough comparative analysis of all available iris segmentation methods is required on reference datasets to pinpoint their effectiveness and limitations. This analysis promotes transparency, reproducibility, and innovation, enabling the identification of gaps in current methodologies and guiding future advancements in the field.

Addressing these gaps, we developed three new open-source models based on U-Net and U-Net++ as benchmarks and created a manual segmentation toolkit along with a manually annotated ground truth dataset that includes eyelash annotations. Additionally, our study performed a comprehensive analysis of eight existing iris segmentation methods—comprising two traditional approaches (OSIRIS [12], USIT (University of Salzburg Iris Toolkit) [32]) and six DL-based approaches ([1,7,18,23,24] selected based on the availability of their source code and the inclusion of eyelash annotations in their ground truths (Table 1). This analysis was conducted using our benchmark models across multiple datasets to evaluate their effectiveness and limitations. Our approach not only addresses current shortcomings in the literature but also establishes a new standard for advancing iris segmentation studies.

## 3. Experiments

Eleven state-of-the-art iris segmentation methods are studied on five datasets for evaluating generalizability, practicability, noise detection capability, and sensitivity towards illumination. In the process, we developed three benchmark iris segmentation models based on U-Net and U-Net++ architecture, prepared a large composite NIR iris dataset by combining multiple public datasets, created a manual iris segmentation toolkit, developed a manually annotated iris-mask ground truth dataset with eyelash annotations for fine-tuning, and adapted eight other algorithms (two traditional and six DL-based) from recent literature, chosen for their accessible source code and ground truth that includes eyelash labels (Table 1). For the purpose of performance evaluation, we focused on studies where the source code or detailed ground truth for iris masks with eyelash annotations was available. Studies lacking in these respects were excluded from our re-evaluation process. The implementation details of all the methods are discussed in this section.

### 3.1. Iris Segmentation Toolkit Development

**Figure 1:**
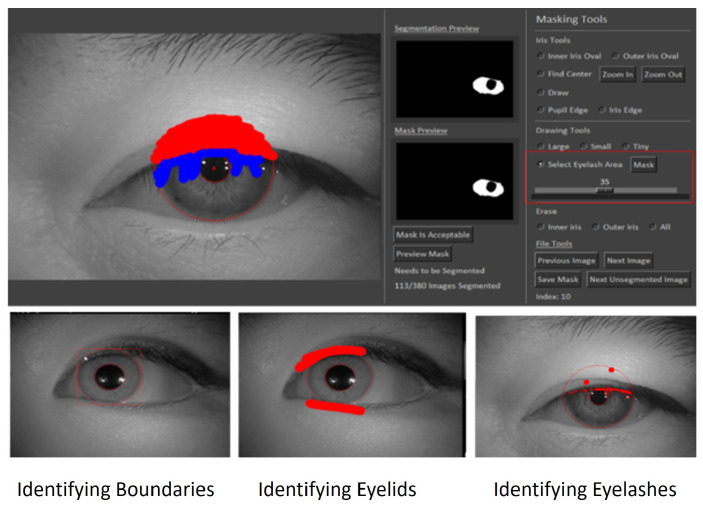
User interface of the iris segmentation toolkit.

**Figure 2:**
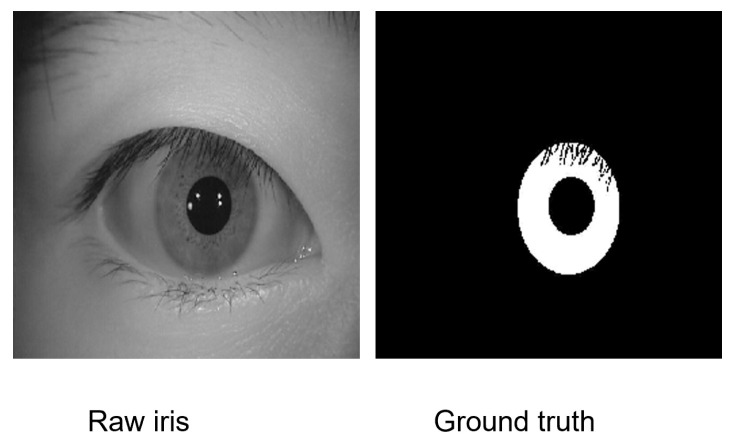
Example of annotated ground truth using the toolkit.

In systems reliant on machine learning, the quality of the input directly influences the quality of the output. Specifically, in the context of iris recognition, the accuracy of iris segmentation and the ability to detect noise are pivotal for the success of the entire recognition process. Thus, for a DL-based iris segmentation model, highly precise ground truth is vital for training. This presents two significant challenges: the scarcity of publicly available, large-scale, high-quality ground truth data, and among the datasets that are available, a limited number include annotations for eyelashes. While OSIRIS, a traditional open-source segmentation tool that employs an integro-differential operator, serves as a resource for generating ground truth, the accuracy of its generated masks is often compromised by challenges such as segmentation errors in cases of low usable iris area, constricted pupils, high illumination, and inaccuracies in eyelash and pupil boundary detection. Our thorough review of existing literature revealed a lack of tools, either manual or automated, dedicated to the generation of iris segmentation masks. Recognizing the critical need for such a resource, we developed a segmentation and annotation toolkit specifically designed for iris segmentation tasks. This toolkit utilizes a pair of ellipses to delineate the iris’s inner and outer boundaries and allows for pixel-level manual annotation to accurately identify eyelids, eyelashes, and other non-iris eye components. The user interface of the toolkit is illustrated in Figure 1. Figure 2 shows an example of annotated ground truth. The toolkit, its features, and the guidance for usage are available in our GitHub repository.

### 3.2. Dataset Preparation

We employ the following five datasets for our experiments. The statistics for each dataset is presented in Table 2.

**(A) Composite dataset:** To address the lack of large annotated iris dataset for the training, we prepared a composite NIR dataset by combining multiple publicly available NIR datasets—CASIA Iris Lamp [25], CASIA Iris Twins [25], CASIA Iris Thousand [25], and ITR Iris Clarkson [33] datasets. Because of the large volume of this dataset, manual segmentation of all the samples would be a daunting task. Instead, Daugman-style open-source OSIRIS [12] was used to generate masks. Segmentation errors by OSIRIS were manually identified and removed. Additionally, VeriEye [34] matcher was used to determine segmentation errors based on the match score (a subset of false accepts and rejects); the errors were removed. The final composite dataset containing 45,683 samples from 1852 subjects is summarized in Table 3. Additionally, we manually annotated 1K samples (referred to as the composite subset dataset for the rest of the paper) using our developed toolkit from the erroneous segmented samples. For visual analysis, we also prepared a separate test set of 60 challenging samples, which OSIRIS failed to segment accurately, leading to a false rejection.

**(B) UBIRIS.v2 NICE.I [28]:** A subset of the UBIRIS.v2 dataset containing manually labeled iris masks used in NICE.I competition [35] was made available by [7]. The subset contains approximately 1K samples, captured on the move and at a distance with a Canon EOS 5D camera under visible illumination at a resolution of 400 × 300 pixels. We utilized the same training and testing set as [7].

**(C) MICHE-I [27]:** MICHE-I was created from 92 subjects by three mobile devices—the iPhone 5, Samsung Galaxy S4, and Samsung Galaxy Tab2—in uncontrolled conditions with visible illumination. A subset of 871 samples from this dataset was compiled by [7] from the original manual annotations by [1,36]. We utilized the training and testing set of [7].

**(D) CASIA Iris Thousand [25]:** We used this entire dataset with the OSIRIS-generated masks to prepare the composite dataset. For our experiment, we also used a subset of 1K samples with manual ground truths shared by [1]. We separately trained all models on these 1K ground truths. Following [1], we utilized 80% for training and 20% for testing.

**(E) CASIA Distance [25]:** CASIA Distance contains 2567 images from 142 subjects captured from a three-meter distance with a CASIA long-range iris camera under NIR illumination. A subset of 400 manually annotated iris masks of resolution 640 × 480 pixels from the first 40 subjects was publicly shared by Liu [37]. Like Wang et al. [7], we used the subset from [37], consisting of 400 iris images with manually labeled iris masks. Following [7], the first 300 images were used for our training, and the last 100 were used for testing.

### 3.3. Iris Segmenter Design

**Figure 3:**
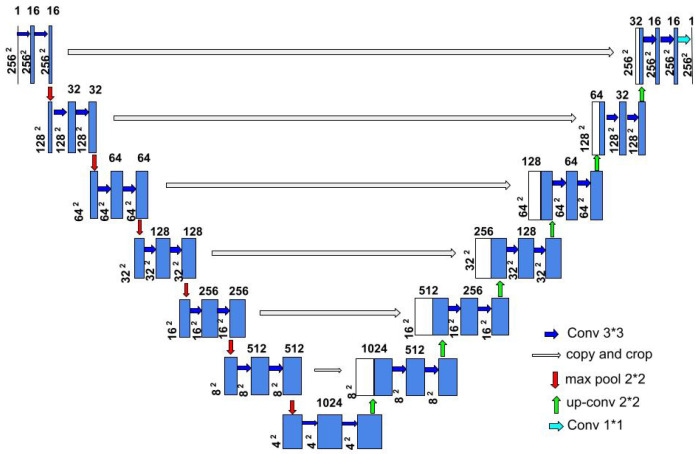
Architecture of the proposed U-Net benchmark model.

**U-Net Architecture**: Our proposed benchmark model, illustrated in Figure 3, is based on a U-Net [13] architecture. The encoder path of this architecture consists of a repeated implementation of two 3 × 3 convolutional layers and a ReLU activation layer with a 2 × 2 max pooling operation with stride 2 for down-sampling. Thus, each down-sampling step doubles the number of feature channels and decreases the image resolution by half. Each step in the decoder path of the architecture consists of a 2 × 2 up-convolution, a concatenation with cropped feature map from the encoder path, and two 3 × 3 convolutions, followed by a ReLU activation layer. Thus, each up-sampling step (opposite to down-sampling) doubles the image resolution and decreases the number of feature channels by half. The concatenation ensures the propagation of all information from the encoder to the decoder and no loss of information while down-sampling. The final layer of the network is a 1 × 1 convolutional layer that combines the preceding layer’s output and produces the segmentation maps.

**Figure 4:**
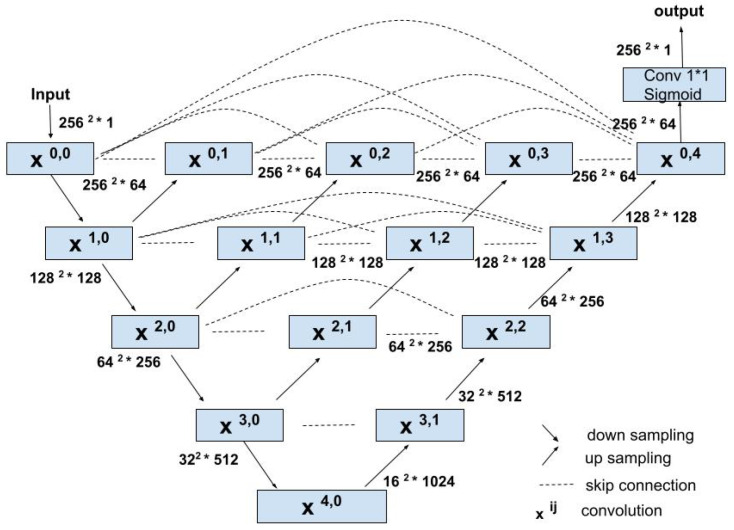
Architecture of the proposed U-Net++ benchmark model.

**U-Net++ Architecture**: Figure 4 shows our second benchmark model based on the U-Net++ architecture [14]. Similar to U-Net architecture, the encoder consists of two repeated 3 × 3 convolution layers, each followed by a ReLU activation layer and a 2 × 2 max-pooling for down-sampling. Unlike the U-Net model, the encoder and decoder are connected through a series of nested dense convolutional blocks, which bridge the semantic gap between the feature maps of the encoder and decoder prior to fusion. Each block in the decoder combines the multiscale feature maps passed horizontally from its preceding blocks (with the same image resolutions) as well as the multiscale feature maps passed vertically (different image resolutions); details are in [14]. Each step in the decoder path also consists of a 2 × 2 up-convolution, concatenation of the preceding multiscale feature map, and two 3 × 3 convolutions, each followed by a ReLU activation layer. Finally, the output of the last decoder passes through a 1 × 1 convolution with sigmoid activation and produces the segmentation mask.

### 3.4. Implementation Details

**U-Net and U-Net++ (ours):** Our proposed benchmark models, illustrated in Figure 3 and Figure 4, are based on U-Net [13] and U-Net++ architecture [14], respectively. We implemented the models in Python using Keras API with TensorFlow as its backend. The training dataset was augmented with rotation, zoom, width, and height shifts to improve the model’s generalization capability. The models are trained with the image resolution of 256 × 256, batch size of 8, Adam optimizer with a learning rate of $10^{-4}$, binary crossentropy loss, and early stopping.

The training procedure was divided into two distinct phases. Initially, the base models, either U-Net or U-Net++, were trained on our composite dataset, which included 45.6k samples (with 90% allocated for training and 10% for validation) using masks generated by the OSIRIS software. This initial phase aimed to establish a robust foundational model. In the subsequent phase, these pre-trained models were fine-tuned on various other datasets to evaluate their adaptability and performance across different imaging conditions and challenges. This included fine-tuning on datasets such as the composite subset (with 1K manually annotated masks), CASIA.v4-Distance [25], CASIA Iris Thousand [25], UBIRIS.v2 NICE.I [28], and MICHE-I [27] datasets, each selected for their unique attributes related to demography, NIR and VIS imaging, sensor types, collection methodologies, and environmental contexts.

The rationale behind this two-step training approach was to first leverage the large volume of data in the composite dataset for foundational training, despite the potential limitations of OSIRIS-generated masks in accurately capturing challenging aspects like eyelashes, eyelids, and eyeglasses, or areas of smaller usable size. The initial training phase potentially limited the models’ performance on such challenging samples. Therefore, fine-tuning the models on manually annotated masks was intended to specifically improve their ability to accurately identify and handle these intricate scenarios, thereby enhancing their overall performance and applicability across a broader range of real-world conditions.

**U-Net with weight map (ours):** In addition to the implementation procedure followed in the U-Net and U-Net++, the U-Net base model was finetuned with the weight-mapped ground truth to increase the weight of the eyelash pixels and draw increased attention to the network to learn eyelash detection, following [13] and weighted binary cross-entropy loss, computed as weightmap×binarycrossentropyloss.

**Lozej et al. [18]:** The authors developed a U-Net-based segmentation method, experimented with different hyperparameter settings, and trained the model on 200 samples (160 samples for training and 40 for testing) from CASIA Iris Interval [25]. The best model weight was made publicly available without the source code. We re-implemented the method following [18]. We used their original pre-trained model as the base model and fine-tuned all the layers independently on five datasets (mentioned above). The authors trained their model for ten epochs. Similarly, Like [18], we trained the model for ten epochs. Additionally, we conducted a separate training using early stopping for a fair comparison.

**Trokielewicz et al. [24]:** The authors developed a postmortem segmentation model based on the SegNet architecture. SegNet consists of an encoder (a pre-trained VGG-16 network excluding the classification layer) and a corresponding decoder, followed by a classification layer. The author initialized the training with pre-trained ImageNet weight [38] and retrained on 1300 manually annotated postmortem iris images obtained from the Warsaw-BioBase-Post-Mortem-Iris v1.0 [29] dataset. The source code is publicly available. We reproduced this method as the authors suggested and fine-tuned it independently on five different datasets (mentioned earlier), initializing with ImageNet weight.

**Bezerra et al. [1]:** The authors proposed two iris segmentation methods based on GAN and FCN. Due to the unavailability of source code, limiting reproducibility, we only report the results presented in the paper. However, the authors made their manually annotated ground truths publicly available. We used their manual ground truths for CASIA Iris Thousand [25] and Miche-I [27] datasets to fine-tune other methods implemented in this study.

**Wang et al. [7]:** The authors proposed a multitask U-Net iris segmentation model, which required an iris mask, pupil mask, and iris outer boundary as the input. The source code is publicly available, including manually annotated pupil masks and outer boundary masks for CASIA Distance [25], UBIRIS.v2 NICE.I [28], and MICHE-I [27] datasets. We did not implement their method in this study as the manual annotation of the pupil mask and iris outer boundary mask for our other test datasets would require additional resources. We reported their performance. We employed their datasets for other implemented methods.

**Wang et al. [23]:** Refs. [7,23] used the same network with a minimum modification. They also used the same two datasets. Hence, we only reported their accuracy.

**The OSIRISv4.1 [12]:** This open-source Dougman-style software comprises four key modules: segmentation, normalization, feature extraction, and matching. We compared the segmentation module with other implementations.

**USIT [32]:** The toolkit includes several traditional iris preprocessing, feature extraction, and feature comparison techniques. We used the Wahet (Weighted Adaptive Hough and Ellipsopolar Transform [39]) segmentation method to compare it with other implemented methods.

## 4. Results

### 4.1. Evaluation Metrics

The following metrics are used to evaluate the segmentation methods.

**mIoU:** Mean Intersection Over Union (mIoU) is a standard evaluation metric for semantic segmentation. IoU indicates the proportion of intersection and union of the ground truth and predicted segmentation. The mIoU is calculated as the mean value of the IoU of all iris images. The mIoU is defined as follows:

(1) $$mIoU=\frac{1}{n}\sum_{i=1}^{n}\frac{\mathrm{True}\;{\mathrm{Positive}}_{i}}{\mathrm{True}\;{\mathrm{Positive}}_{i}+\mathrm{False}\;{\mathrm{Positive}}_{i}+\mathrm{False}\;{\mathrm{Negative}}_{i}}$$

**F1 score:** F1 score is the harmonic mean of the precision and recall. Precision is the ratio of the number of correctly classified iris pixels to the number of all pixels that are classified as iris pixels, including those not identified correctly. The recall is the ratio of the number of correctly classified iris pixels to the number of all iris pixels that should have been identified as the iris. F1 is calculated as follows:

(2) $$F1\;\mathrm{score}=\frac{2\times \mathrm{True}\;\mathrm{Positive}}{2\times \mathrm{True}\;\mathrm{Positive}+\mathrm{False}\;\mathrm{Positive}+\mathrm{False}\;\mathrm{Negative}}$$

F1 and mIoU scores are bounded between 0 and 1. A higher score represents better segmentation accuracy. Both the F1 score and mIoU are widely recognized and standard metrics in the field of iris segmentation for evaluating model accuracy, providing a reliable assessment of segmentation performance in biometric systems.

### 4.2. Performance Evaluation

We conducted a comprehensive quantitative and qualitative analysis of 11 state-of-the-art segmentation models, including the three benchmark models we developed. This thorough analysis involved intra-dataset performance assessments across five diverse datasets, taking into account various factors such as demography, NIR and VIS illumination, sensor types, collection setups, and environmental conditions. Furthermore, we assessed the models’ ability to detect noise (eyelash detection), their cross-dataset performance, and practical usability.

In addition to intra-dataset performance, we placed emphasis on cross-dataset evaluation to assess the generalization capability of the models. This is a crucial aspect for biometric systems that need to operate effectively on unseen data in real-world applications. Our benchmarking methodology also accounts for practicability by evaluating inference time and computational efficiency, both of which are essential for real-time deployment in biometric systems. These factors together ensure that the models not only excel in accuracy but are also robust and practical for deployment.

**(A) Intra-dataset performance evaluation:** a detailed summary of intra-dataset performance is provided in Table 4, with significant insights for each dataset detailed below:

**Composite subset (NIR):** Our benchmark model, U-Net, achieved the highest mIoU score of 91.70% and the second-highest F1 score of 94.04%. Our U-Net++ model secured the second-highest mIoU of 91.41% and the highest F1 score of 94.20%. Lozej et al. (FT-10) and Lozej et al. (FT-ES) posted mIoU scores of 91.13% and 91.16%, with F1 scores of 93.62% and 94.01%, respectively. Our U-Net with weight map model achieved 91.03% mIoU and 93.80% F1 score. Trokielewicz et al. (FT), OSIRIS, and USIT performed with mIoU scores of 85.09%, 85.98%, and 87.30%, and F1 scores of 88.14%, 87.17%, and 87.10%, respectively. This indicates that these models are less suited for this dataset compared with the other DL-based models.

**CASIA Thousand (NIR):** Our U-Net++ models achieved the highest mIoU score of 95.26% and the second-highest F1 score of 95.22%. Our U-Net model also demonstrated comparable results. The models developed by Lozej et al. achieved mIoU scores similar to those of U-Net and U-Net++, albeit with a lower F1 score of 94.61%. The FCN and GAN models by Bezerra showed robust performance, with F1 scores of 94.42% and 95.38% (highest among all models), respectively, suggesting they are highly effective for this dataset. The performance of Trokielewicz et al. (FT) and traditional methods such as OSIRIS v4.1 and USIT (Wahet) was comparatively degraded, with IoU scores of 82.98%, 88.51%, and 80.83%, and F1 scores of 89.52%, 87.78%, and 81.62%, respectively.

**CASIA Distance (NIR):** Our U-Net++ model achieved the highest IoU and F1 scores of 94.72% and 94.51%, respectively. The U-Net model achieved a mIoU of 94.48% and an F1 score of 93.58%. The models of Lozej et al. (FT-10) and Lozej et al. (FT-ES) secure mIoU scores of 92.80% and 93.56% and F1 scores of 92.20% and 93.04%, respectively. Models developed by Wang [7] and Wang [23] both achieved F1 scores of 94.25% and 94.30%, respectively. However, Wang’s [23] model achieved a lower mIoU score (89.40%) compared with our models and Lozej’s models. The model from Trokielewicz et al. (FT) recorded an IoU of 79.38% and an F1 score of 85.28%, highlighting its lesser effectiveness compared with the other deep learning (DL) models. The traditional method, OSIRIS v4.1, achieved an IoU of 83.38% and an F1 score of 82.87%, demonstrating that traditional approaches are less effective for this dataset in comparison to DL-based models. USIT (Wahet) achieved a mIoU of 70.34% and an F1 score of 72.45%, indicating its unsuitability for this dataset and further underscoring the superiority of DL-based methods for this dataset.

**UBIRIS.v2 NICE.I (VIS):** Our U-Net and U-Net++ models, along with Lozej’s models, achieved F1 scores of 90.89%, 90.87%, and 90.66%, respectively. In terms of the IoU scores, our U-Net++ and U-Net models recorded the highest (91.86%) and second highest (91.78%) scores, respectively, while Lozej’s model achieved a mIoU of 91.04%. Wang et al. reported the highest F1 score of 91.78% for this dataset, indicating top performance among the evaluated models. Additionally, Bezerra’s GAN demonstrated strong performance with an F1 score of 91.42%. The performance of Bezerra’s FCN, with an F1 score of 88.20%, and the Trokielewicz et al. (FT) models, with an F1 score of 85.28%, was less effective in segmentation compared with other deep learning models for this dataset. Traditional methods, such as OSIRIS v4.1 and USIT (Wahet), were found to be unsuitable for this dataset, achieving mIoU and F1 scores ranging from 20% to 43%, further underscoring the inadequacy of traditional methods for this dataset.

**MICHE-I (VIS):** Our benchmark models U-Net and U-Net++ stood out with the highest and second-highest mIoU scores of 92.98% and 92.94%, respectively, complemented by F1 scores of 92.27% and 92.82%. Lozej’s model achieved a mIoU of 92.36% and a F1 score of 91.83%. Trokielewicz’s model was less effective, achieving an IoU score of 84.21% and an F1 score of 83.42%. Bezerra’s FCN and GAN models showed F1 scores of 83.03% and 87.20%, indicating they were less efficient than the U-Net variants. Traditional methods, OSIRIS v4.1 and USIT (Wahet), showed comparatively degraded performance, with F1 scores of 32.48% and 26.03%, respectively, underscoring their unsuitability for this dataset.

In summary, the performance of all U-Net variant models, including those developed by us, Lozej, and Wang, was comparable and consistent across both NIR and VIS datasets, with minimal differences in accuracy observed. The FCN model demonstrated strong performance on the CASIA Thousand dataset but was less effective on the UBIRIS.v2 NICE.I (VIS) and MICHE-I (VIS) datasets, indicating its reduced efficacy for VIS datasets. Similarly, GAN-based models exhibited robust performance for the CASIA Thousand and UBIRIS.v2 NICE.I (VIS) datasets but were less effective on the MICHE-I (VIS) dataset compared with the U-Net variants. DL-based iris segmentation methods, with the exception of the model developed by Trokielewicz et al. [24], consistently outperformed traditional methods such as OSIRIS [12] and USIT [32] across all evaluated metrics for each dataset. Trokielewicz et al. [24] surpassed traditional methods in all metrics on VIS datasets and achieved higher F1 scores, albeit with a lower mIoU score than OSIRIS, for NIR datasets. Initially designed for postmortem data, the model by Trokielewicz et al. [24] notably outperformed OSIRIS on postmortem datasets. However, our observations lead to the conclusion that a model tailored for postmortem iris analysis can not be optimally fine-tuned for live iris datasets, resulting in its under-performance compared with more recent DL-based models and traditional iris segmentation methods on NIR datasets with live subjects. These findings further underscore that traditional methods are not well-suited for VIS datasets.

**(B) Assessment of eyelash detection capability:** To evaluate the models’ capability in eyelash detection, we separated 160 samples with eyelash occlusions from the test set of the composite subset dataset (refer to mIoU EL in Table 4). mIoU was used as an evaluation metric. mIoU refers to the score for the entire test set of 330 samples. Our three models and Lozej’s model demonstrated strong (>90%) accuracy and comparable performance in eyelash occlusion detection. Despite these high accuracy rates, a decline in performance was noted for the models when dealing with samples containing eyelash occlusions, as opposed to their performance on the complete general test set. Limited training iris masks with annotated eyelashes could be the reason for the lower scores. Adding more training masks with annotated eyelashes may improve this performance.

**(C) Cross-dataset performance evaluation:** Generalization capability is a cornerstone of biometric recognition systems, ensuring that models can effectively adapt to and classify new, unseen data. To assess the generalization capability of the implemented models, we conducted cross-dataset testing. The models, fine-tuned on the composite subset dataset, were tested on the CASIA Distance dataset without any further tuning. The CASIA Distance dataset was not included in our composite or composite subset dataset. Its images were captured from a three-meter distance under moving conditions with NIR illumination, making it distinct from the other CASIA datasets and unseen by the models trained on the composite or composite subset dataset. The cross-dataset performance is shown in Table 5.

Table 5 indicates that all of our proposed models exhibited strong generalization capabilities and outperformed all the implemented methods. The U-Net model achieved a mIoU of 0.9231 and an F1 score of 91.43%, while the U-Net++ model showed a slight improvement with a mIoU of 0.9331 and an F1 score of 92.01%. These results establish a standard benchmark for generalization capability in iris segmentation models. Conversely, the original model from Lozej et al. showed limited adaptability, with a mIoU of 0.4347 and an F1 score of 6.67% when applied to the CASIA Distance dataset. However, once fine-tuned on our composite subset dataset, the same model’s performance was significantly enhanced, reaching a mIoU of 0.9080 and an F1 score of 89.72%. This demonstrates that even models initially lacking in generalization can achieve benchmark standards with the appropriate training dataset. Similarly, Trokielewicz et al.’s original model was outperformed by our benchmark models when tested on the CASIA Distance dataset. It performed with a mIoU of 0.7398 and an F1 score of 79.28%. After fine-tuning, there was a notable increase to a mIoU of 0.8137 and an F1 score of 87.91%, though this was still below the performance of our benchmark models. Overall, all the current open-source DL segmentation models have limited generalization capability. These results underscore the importance of comprehensive and diverse training in the development of models that excel not only in familiar conditions but also maintain high accuracy when faced with new and challenging datasets. Our benchmark models serve as a robust standard for the future development of the field of iris segmentation, guiding researchers toward creating more adaptive and reliable biometric recognition systems.

**(D) Assessment of practicability:** The practicability of the DL models is evaluated in terms of the number of parameters, storage space, and inference time (the duration required for a single prediction) of all models, as summarized in Table 6. These factors are critical for the deployment of models in practical applications, where efficiency and minimal resource consumption are often as important as accuracy. We measured the average inference time on a desktop configured with an Intel Core i9-12900K CPU, evaluating .h5 models. Our evaluation indicates the Wang et al. model requires the lowest storage space at 100 Mb, while Lojez’s model requires 13 times more space at 13.8 GB. The Trokielewicz model has the fastest inference time at 0.004 s with a storage space of 104 Mb. In contrast, Lojez’s model requires the highest inference time of 0.294 s. Our U-Net and U-Net with weighted map models have comparable storage space (360 MB) and inference time (0.02 s). Our U-Net++ model requires a storage space of 414 Mb and an inference time of 0.269 s. Overall, all models present significant computational and storage demands, making their implementation on mobile devices challenging. Therefore, further optimization of these models is required.

**Figure 5:**
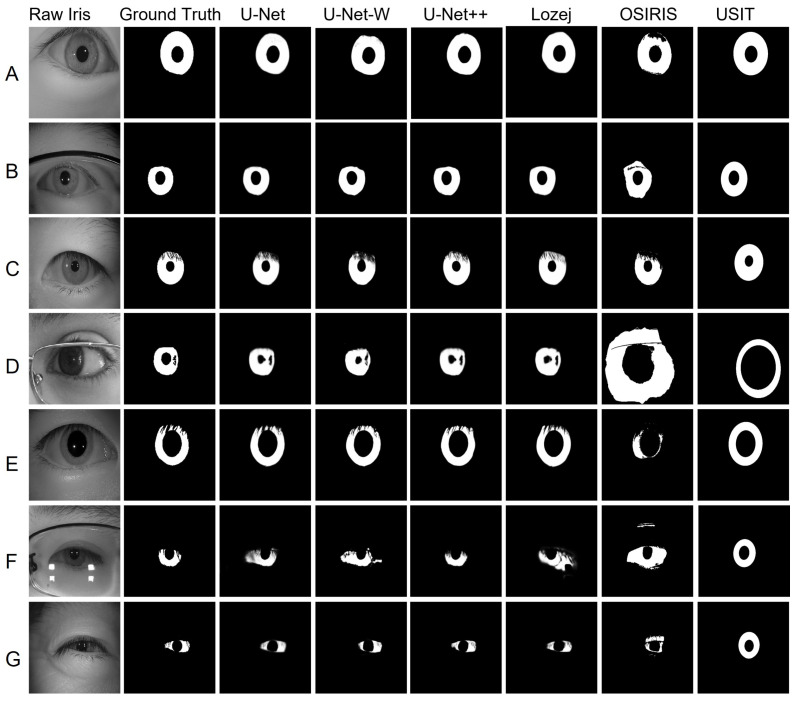
Examples of segmentation outputs from different models across various cases: (**A**) Iris without occlusions, (**B**) Iris with eyeglass occlusion, (**C**) Iris with eyelash occlusion, (**D**) Off-angle iris with eyeglass occlusion and specular reflection, (**E**) Eyelash occlusion with a dilated pupil, (**F**) Eyeglass occlusion and specular reflection, (**G**) Smaller iris area.

**(E) Visual evaluation:** Figure 5 shows the segmentation results of the implemented methods from different datasets. Generally, noise such as eyelashes, eyelid occlusion, specular reflections, uneven illumination, off-angle iris, and smaller-sized iris make the segmentation task difficult. To evaluate all models’ performance on those edge cases, we visually assessed all challenging iris images from all the datasets. The findings are summarized here:

- DL methods showed improved noise masking capability compared with the traditional methods (OSIRIS and USIT) for cases like eyelid and eyelash occlusions, high and low illuminated samples, variable pupil dilation, smaller iris area, eyeglasses, and off-angle iris.
- A few incorrect segmentations were observed for cases with eyeglasses, eyelash occlusion, and off-angle iris samples. Limited training samples representing these cases might be the cause of these errors. Hence, adding more annotated training images with those cases may improve the segmentation performance.
- OSIRIS showed limited capability in eyeglass and eyelash detection.
- USIT could not detect eyelashes.
- Both OSIRIS and USIT performed poorly against off-angle iris.

Note: For visual comparison, we only compared OSIRIS and USIT’s performance on the NIR dataset, as they were unsuitable for VIS datasets.

## 5. Discussion and Conclusions

In this study, we conducted a comprehensive review and benchmarked eleven iris segmentation models across five different datasets (including NIR and VIS) to evaluate their effectiveness and limitations. In the process, we prepared an end-to-end iris segmentation resource set comprising a large training dataset (gleaned from multiple public sources), developed an iris segmentation toolkit, and manually annotated 1k images to address the unavailability of iris masks with eyelash labels. We developed three and adapted eight algorithms for comparison (two traditional and six DL-based).

Though NIR is predominantly utilized for iris recognition, there is a persistent interest [3,4] in the community to enhance VIS-based iris recognition for their widespread adaptability and less stringent hardware requirements. To assess the viability of VIS-based recognition, we incorporated two VIS-based datasets into our evaluation. Our evaluation demonstrates that DL models can adapt to VIS-based datasets and achieve comparable performance to NIR-based datasets upon fine-tuning, while traditional methods fail under these conditions. These efforts in research are driven by the desire to broaden the applications of VIS-based iris recognition, particularly for its potential to reduce hardware complexities associated with NIR collection, thus facilitating integration into portable devices like smartphones.

Our experimental findings demonstrate that Bezerra’s FCN models perform well in the NIR dataset while showing less effectiveness in VIS datasets, especially in the MICHE-I dataset. This discrepancy is likely due to the greater variability in visible light images, which can include differences in lighting conditions, colors, and contrasts that are not as prevalent in NIR images. This suggests the need for improved models or training strategies that can better handle the diversity in VIS data. Similarly, the mixed success of Bezerra’s GAN-based models across datasets underscores the requirement of careful adjustment to their architecture or training protocols to enhance their performance across a diverse range of real-world scenarios. Additionally, our analysis highlighted the consistent performance of all U-Net architecture-based models (developed by us, Lojez, and Wang) across both NIR and VIS datasets, demonstrating their flexibility under varying imaging conditions. However, the observation of marginal accuracy improvements among these DL models within specific datasets indicates a point of diminishing returns in focusing solely on architectural advancements. This suggests that augmenting the variety and representativeness of training datasets might offer more significant benefits, encouraging models to better capture and respond to the complexities encountered in practical applications.

The comparison with traditional approaches demonstrates that deep learning (DL) models show improvement over traditional methods for challenging samples, such as eyelid and eyelash occlusions, eyeglasses, high and low illuminated samples, variable pupil dilation, smaller iris areas, and off-angle irises. However, there is still scope for improvement in eyelash detection, eyeglasses, and off-angle iris segmentation. The consistent outperformance of DL models over traditional methods across all metrics and datasets reaffirms the transformative impact of DL in biometric recognition. However, Trokielewicz et al.’s [24] SegNet-based model, which is efficient in memory and inference time and designed for postmortem iris segmentation, demonstrates the trade-off between specialization and generalization. Its focus on postmortem data leads to reduced effectiveness on live samples while finetuning on live iris datasets, with accuracy dropping by 6–15% in mIoU and 6–9% in F1 scores compared with other DL approaches (Table 4). This underscores the importance of developing versatile models that maintain high performance across both live and specialized scenarios, highlighting the need for a balance between model specialization and general applicability in biometric recognition.

The proposed U-Net benchmark architecture is a balanced model showing comparable or better performance with high cross-dataset performance (only outperformed by our U-Net++) high eyelash detection capability (only surpassed by Lozej) with an inference time of 0.02 s (bested only by Trokielewicz) and a storage space of 360 Mb. Lozej’s model shows comparable performance to our proposed U-Net model in certain datasets, but it falls short in generalization capability and is significantly less efficient, requiring 13 times more storage and a 14 times higher inference time.

Increasing the weight of the eyelashes to train the U-Net with the weight map model did not improve the accuracy. The dense convolution blocks in the skip connection of U-Net++ increased the computation and hardware cost of the network. It showed comparable overall performance with the U-Net (refer Table 4). However, the impact is seen in our generalizability assessment, where it outperforms all models by a considerable margin.

Wang et al. [7] performed an ablation study and showed multi-tasking U-Net (trained on the iris mask, pupil mask, and iris outer boundary mask) performed better than their U-Net (trained on only the iris mask). Adding an attention module with ASPP (Atrous Spatial Pyramid Pooling) improved the model’s performance (0.23% on Miche-I and 0.89% on UBIRIS) compared with our models. However, annotation of the pupil mask and iris outer boundary mask needed more effort. Incorporating attention modules with ASPP (Atrous Spatial Pyramid Pooling), as proposed by Wang et al. [7], into our framework could potentially enhance our model’s accuracy by capturing multi-scale contextual information and focusing on the most important features while suppressing irrelevant noise. ASPP, integrated within the attention module, enables the model to process both local and long-range spatial features. This may improve segmentation performance, particularly in challenging cases involving occlusions from eyelashes, eyeglasses, or other distractions. Additionally, the integration of ASPP within the attention mechanism could help address the scale-sensitivity problem, potentially allowing the model to detect iris features across varying scales, including images with different iris sizes and pupil dilation.

Our benchmark models, achieving high mIoU and F1 scores on the cross dataset (Table 5), set a new standard in the field, offering a robust framework for evaluating the generalization capabilities of future models. Current open-source models exhibit limited generalization capabilities and lag behind our benchmarked standards even after finetuning on our composite subset datasets (Table 5). This sets a challenge for the community to advance model performance and develop more adaptable, reliable biometric systems.

DL models often require substantial computational and storage resources. It’s essential to develop more efficient DL networks to ensure their practicality for widespread use. Therefore, further optimization of existing benchmark models remains an important area for future research. Additionally, future research should address gaps in eyelash detection eyeglasses and off-angle iris segmentation, considering the trade-offs between model complexity and data availability. This evaluation showcases the critical role of training data diversity and model generalization, with models like Lozej et al. showing significant improvement after fine-tuning with diverse data. This underscores the need for innovative architectures and training datasets that are diverse and representative. A limitation of our research is the relatively small size of the fine-tuning datasets, each containing around 1000 samples. This was primarily due to the scarcity of annotated iris images with eyelash labels. Our open-source benchmark models, iris segmentation toolkit, and a manually annotated dataset of 1K iris masks will support and encourage further research in this field.

## Figures and Tables

**Table 1:** The state-of-the-art iris segmentation algorithms.

Research	Resource Availability			
	**Code**	**Training Mask**	**Test Ground Truth**	**Eyelash Marking**
Miron et al. [15]				
Hou et al. [8]				
Yang et al. [16]				
Meng et al. [17]				
Lozej et al. [18]	✓			✓
Zang et al. [19]		✓		
Jalilian et al. [20]		✓		
Li et al. [21]		✓	✓	
Chen et al. [22]		✓		
Wang et al. [7]	✓	✓	✓	✓
Wang et al. [16]				
Wang et al. [23]		✓	✓	✓
Bezerra [1]		✓	✓	✓
Trokielewicz et al. [24]	✓	✓		
Proposed work	✓	✓	✓	✓

**Table 2:** Summary of all datasets used in this study.

Dataset	Total Images	Train Samples	Test Samples	Resolution	Wavelength
Composite	45,683	41,283	4400	640 × 480	NIR
Composite Subset	1006	616	330	640 × 480	NIR
CASIA Distance	400	300	100	640 × 480	NIR
CASIA Thousand	1000	800	200	640× 480	NIR
UBIRIS.v2NICE.I	915	479	428	400 × 300	VIS
MICHE-I	871	680	191	Various	VIS

**Table 3:** An overview of the composite dataset.

Dataset	No of Subjects	Unique ID	No of Samples
CASIA Iris Thousand	1000	2000	19,338
CASIA Iris Twin	200	400	3082
CASIA Iris Lamp	410	820	16,081
ITR Clarkson	242	479	7182
Total	1852	3699	45,683

**Table 4:** Performance evaluation of the state-of-the-art models on five benchmarking datasets.

Method	Composite Subset (NIR)					Casia Thousand (NIR)			Casia Distance (NIR)			UBIRISv2NICE.I (VIS)			MICHE-I (VIS)		
	**Th**	**mIoU (0-1)**	**F1 (%)**	**mIoU EL**	**F1 (%) EL**	**Th**	**mIoU (0-1)**	**F1 (%)**	**Th**	**mIoU (0-1)**	**F1 (%)**	**Th**	**mIoU (0-1)**	**F1 (%)**	**Th**	**mIoU (0-1)**	**F1 (%)**
U-Net (our)	0.75	**0.9170**	**94.03**	**0.9083**	93.27	0.65	**0.9508**	95.07	0.3	**0.9448**	93.58	0.5	**0.9178**	90.89	0.5	**0.9298**	92.27
U-Net-W (our)	0.75	0.9103	93.80	0.9001	92.79	0.65	0.9465	94.57	0.25	0.9344	92.41	0.5	0.9057	89.20	0.35	0.9170	90.67
U-Net++ (our)	0.75	**0.9141**	**94.20**	0.9064	**93.85**	0.60	**0.9526**	**95.22**	0.2	**0.9472**	**94.51**	0.45	**0.9186**	90.87	0.45	**0.9294**	**92.82**
Lozej [18] (FT-10)	0.7	0.9113	93.62	**0.9088**	**93.33**	0.75	0.9488	94.61	0.40	0.9280	92.20	0.45	0.9048	89.77	0.4	0.9163	90.90
Lozej [18] (FT-ES)	0.8	0.9116	94.01	0.9065	93.30	0.65	0.9506	94.67	0.25	0.9356	93.04	0.45	0.9104	90.66	0.45	0.9236	91.83
Trok [24] (FT)	n/a	0.8509	88.14	0.8520	88.38	n/a	0.8298	89.52	n/a	0.7938	85.28	n/a	0.8524	85.28	n/a	0.8421	83.42
Wang [7]	n/a	n/a	n/a	n/a	n/a	n/a	n/a	n/a	n/a	n/a	94.25	n/a	n/a	**91.78**	n/a	n/a	**93.05**
Wang [23]	n/a	n/a	n/a	n/a	n/a	n/a	n/a	n/a	n/a	89.40	**94.30**	n/a	84.79	91.33	n/a	n/a	n/a
Bezerra [1] (FCN)	n/a	n/a	n/a	n/a	n/a	n/a	n/a	94.42	n/a	n/a	n/a	n/a	n/a	88.20	n/a	n/a	83.03
Bezerra [1] (GAN)	n/a	n/a	n/a	n/a	n/a	n/a	n/a	**95.38**	n/a	n/a	n/a	n/a	n/a	**91.42**	n/a	n/a	87.42
OSIRIS v 4.1	n/a	0.8598	87.17	0.8547	87.81	n/a	0.8851	87.78	n/a	0.8338	82.87	n/a	0.4327	20.97	n/a	0.4911	32.48
USIT (Wahet)	n/a	0.8730	87.10	0.8601	85.36	n/a	0.8083	81.62	n/a	0.7034	72.45	n/a	0.3448	20.94	n/a	0.4058	26.03

Annotations: Th: Threshold, EL: sample with eyelash occlusion, U-Net-W: U-Net with weight map, Trok (FT): Trokielewicz [24] (fine-tuned), FT-10: fine-tuned for 10 epoch, FT-ES: fine-tuned using early stopping. All methods are trained or tuned independently on the five training datasets and then evaluated on the respective testing set for fair comparisons. Bold values indicate the top two highest accuracy results for each metric across the different segmentation models.

**Table 5:** Cross-dataset performance (Test DB: CASIA Distance).

Method	Train DB	Th	mIoU	F1 Score (%)
U-Net (ours)	Composite	0.6	0.9231	91.43
U-Net++ (ours)	Composite	0.75	**0.9331**	**92.01**
U-Net with weight map (ours)	Composite	0.75	0.9150	89.72
Lozej [18] (original)	CASIA Interval	0.80	0.4347	6.67
Lozej [18] (fine-tuned)	Composite	0.65	0.9080	89.72
Trokielewicz [24] (original)	Postmortem	n/a	0.7398	79.28
Trokielewicz [24] (fine-tuned)	Composite	n/a	0.8137	87.91

Note: Bold values highlight the best accuracy results across the models for each metric.

**Table 6:** An overview of deep learning models assessed in this study.

Research	Architecture	Input Image Resolution	Resource Availability		Eyelash Detection	No of Parameter	Storage Space	Average Inference Time
			**Code**	**Ground Truth**				
U-Net (ours)	U-Net	256 × 256	✓	✓	✓	31.46 M	360 MB	0.021 s
U-Net++ (ours)	U-Net++	256 × 256	✓	✓	✓	36.16 M	414 MB	0.269 s
U-Net-W (ours)	U-Net	256 × 256	✓	✓	✓	31.46 M	360 MB	0.024 s
Lozej et al. [18]	U-Net	320 × 320	✓		✓	124.36 M	1.38 GB	0.294 s
Trokielewicz et al. [24]	Seg-Net	160 × 120	✓	✓		29.4 M	104.65 MB	0.004s
Wang et al. [7]	Multi Tasking U-Net	n/a	✓	✓	✓	31.28 M	119 MB	n/a
Wang et al. [23]	Multi Tasking U-Net	n/a		✓	✓	n/a	100 MB	n/a
Bezerra [1]	FCN and GAN	512 × 512		✓	✓	n/a	n/a	n/a

## Data Availability

The required datasets for the experiment can be obtained from: 1. Clarkson: https://tinyurl.com/43euw7ca (accessed on 24 February 2024). 2. CASIA: http://www.idealtest.org/dbDetailForUser.do?id=4 (accessed on 24 February 2024). 3. MICHE-I Email: biplab@unisa.it. 4. UBIRIS.v2 Email: hugomcp@di.ubi.pt.

## Glossary

**Term**	**Definition**
Iris segmentation	The process of isolating the iris from the rest of the eye in an image, including separating it from other structures like the eyelid, eyelashes, and surrounding areas.
Deep Learning (DL)	A subset of machine learning involving neural networks with multiple layers, used for tasks such as image segmentation and classification.
U-Net/U-Net++	Specific deep learning architectures designed for image segmentation tasks. U-Net++ enhances U-Net by adding nested convolutional blocks to improve feature representation.
Cross-dataset performance analysis	Evaluating a model’s ability to perform well on datasets that were not used during its training phase, important for testing model generalization.
Generalization capability	The ability of a model to apply what it has learned from one dataset to new, unseen data from different datasets.
mIoU (Mean Intersection Over Union)	A metric used to evaluate the accuracy of segmentation models by comparing the overlap between predicted and ground truth areas.
F1 Score	A measure of a model’s accuracy that considers both precision and recall, especially in binary classification tasks like pixel-wise segmentation.
NIR (Near-Infrared)	A part of the light spectrum used in iris imaging, typically in the wavelength range of 700 to 900 nanometers (nm), for capturing iris patterns under controlled lighting conditions.
VIS (Visible Spectrum)	The portion of the electromagnetic spectrum visible to the human eye, ranging from 380 to 720 nanometers (nm). While the use of VIS for iris recognition is still an area of ongoing research, it holds potential to reduce hardware complexities associated with NIR collection, making it easier to integrate iris recognition into portable devices such as smartphones.
Benchmarking	The process of comparing different models or algorithms using specific metrics across common datasets to evaluate their performance.
Eyeglash occlusion	A condition where parts of the iris image are blocked by eyeglashes, making segmentation more difficult.
Practicability	A measure of how feasible it is to implement a model in real-world applications, considering factors like inference time and computational requirements.
Inference time	The time a model takes to make a prediction, critical for real-time applications.
Ground truth	Manually labeled data used as a standard or reference for evaluating the performance of algorithms, such as manually segmented iris masks.
OSIRIS	An open-source iris recognition software that provides a complete iris recognition pipeline, including segmentation, feature extraction, and matching.
USIT	An iris recognition software that provides a complete open-source framework for iris segmentation, feature extraction, and matching, often used in biometric research alongside OSIRIS.
Composite dataset	A dataset created by combining samples from multiple publicly available datasets to form a larger, more diverse set of images for training or evaluation.
Noise detection capability	The ability of a segmentation algorithm to identify and handle artifacts like reflections or occlusions that can interfere with the accuracy of the iris segmentation process.
Specular reflection	The bright spot or glare in an image caused by light reflecting off curved surfaces, such as the eye or eyeglasses. This reflection can obscure iris details and negatively impact segmentation accuracy.
Dilation	The process by which the pupil expands in response to low light or other stimuli. Pupil dilation can alter the visible area of the iris, potentially impacting segmentation accuracy.
